# Prognosis and determinants of serum PTH changes over time in 1-5 CKD stage patients followed in tertiary care

**DOI:** 10.1371/journal.pone.0202417

**Published:** 2018-08-23

**Authors:** Silvio Borrelli, Paolo Chiodini, Luca De Nicola, Roberto Minutolo, Michele Provenzano, Carlo Garofalo, Giuseppe Remuzzi, Claudio Ronco, Mario Gennaro Cozzolino, Carlo Manno, Anna Maria Costanzo, Giuliana Gualberti, Giuseppe Conte

**Affiliations:** 1 Division of Nephrology, University of Campania "Luigi Vanvitelli, Naples, Italy; 2 Medical Statistics Unit, University of Campania "Luigi Vanvitelli", Naples, Italy; 3 IRCCS—Istituto di Ricerche Farmacologiche Mario Negri, Centro Anna Maria Astori, Science and Technology Park Kilometro Rosso, Bergamo, Italy; 4 Unit of Nephrology, Azienda Socio-Sanitaria Territoriale (ASST) Papa Giovanni XXIII, Bergamo, Italy; 5 Department of Biomedical and Clinical Sciences, University of Milan, Milan, Italy; 6 Department of Nephrology, Dialysis and Transplantation, International Renal Research Institute of Vicenza (IRRIV) San Bortolo Hospital, Vicenza–Italy; 7 Department of Health Sciences, Renal Division, ASST Santi Paolo e Carlo, University of Milan, Italy; 8 Unità Operativa Complessa Nefrologia, Dialisi e Trapianti, Dipartimento Emergenza e Trapianti d’Organo, Università degli Studi “Aldo Moro”, Bari, Italy; 9 AbbVie, Campoverde (LT), Italy; Universidade Estadual Paulista Julio de Mesquita Filho, BRAZIL

## Abstract

International Guidelines for mineral bone disorders recommend that in Non Dialytic-Chronic Kidney Disease (ND-CKD) clinical decisions should be based on the trend of serum PTH changes over time rather than on a single value. However, the prognostic impact of these changes in ND-CKD patients remains unknown. We performed a multicenter cohort study in ND-CKD patients (stage 1–5) followed for 36 months in 24 Italian Nephrology Units. PTH changes (ΔPTH) were defined as the absolute differences between all available PTH measurements following the first control and basal value. Primary endpoint in this subanalysis was renal death (End-Stage Renal Disease (ESRD) or all-causes death before ESRD). Association between renal death and ΔPTH was assessed by time-dependent Cox model for repeated measurements. Out of the original cohort (N = 884), we selected 543 patients (66.3±15.4 ys, 58.4% males) with at least two serum PTH measurements. At baseline, eGFR was 36 (IQR: 22.4–56.8) mL/min/1.73m^2^ and serum PTH 46 (IQR: 28–81) pg/mL. ΔPTH was in median 0 (IQR:-18/18) pg/mL. Basal predictors of longitudinal PTH increments were higher serum phosphate, more advanced CKD stages and lower serum PTH. Fully adjusted Cox model with ΔPTH quartiles as discrete time-dependent covariate showed a significant risk of renal death in the highest quartile (HR: 1.91; 95%CI:1.08–3.38; P = 0.026). Considering ΔPTH, as continuous time-dependent variable, (HR:1.02; 95%C.I.: 1.01–1.04; P = 0.004), risk of renal death progressively rose as ΔPTH increased. An increment in serum PTH over time is associated with a worse prognosis in ND-CKD patients, independently from baseline or any absolute concentration of serum PTH and phosphate.

## Introduction

In non-dialysis chronic kidney disease (ND-CKD), serum levels of parathyroid hormone (PTH) progressively increase since the early stages of disease in order to preserve phosphate (P) homeostasis [1]. Indeed, nearly 20% of patients with GFR>60 mL/min/1.73m^2^ have elevated PTH levels that further increase as renal function deteriorates [2–4]. This adaptive mechanism modifies renal prognosis as testified by several studies showing a strong association between high PTH and the risk of faster renal progression [5–8].

Current guidelines, recently updated [9] suggest that medical management of CKD-dependent Mineral Bone Disease (CKD-MBD) should be based on serial measurements of serum PTH and also therapeutic decisions should be based on trend rather than on a single laboratory value. However, this recommendation remains opinion-based being not supported by any clinical study assessing changes over time of calcium, phosphate or PTH. This is critical information, considering that fluctuations over time are expected due to either worsening of renal function or therapeutic interventions, such as dietary phosphate and protein intake restriction, vitamin D, calcium salts and phosphate binders. It is also important to highlight that the prognostic studies so far available in ND-CKD patients have examined associations between secondary hyperparathyroidism and adverse outcome exclusively based on the baseline serum measurements of MBD parameters and, therefore, not accounting for the changes over time [5–8].

To fill this gap of knowledge, we performed a prospective analysis in a cohort of patients with stages 1–5 CKD, regularly followed for 36 months in 24 Italian Nephrology units, and evaluated the prognostic effect of PTH change over time.

## Methods

### Study design

This is a multicenter observational prospective cohort study (IRIDE study) performed in ND-CKD patients afferent to 24 Italian Nephrology units from December 2010 to September 2014. The sample of patients afferent from each participating centre was extremely variable, ranged from 3 to 45 (median:30). The study was approved by Institutional Review Board and Ethical Committee of each participating center; according to local national regulations (first Ethics Committee approval was granted by the local Ethics Committee of “Azienda USL 10 di Firenze” on September the 9^th^ 2010). Patients gave written consent to use their clinical data.

We enrolled adult patients affected by CKD from stage 1 to stage 5, according to criteria of KDIGO Guidelines [10], followed in the clinic from at least six months. Exclusion criteria were advanced malignancy, advanced liver or heart failure and renal transplantation.

As described in previous papers [11,12], the sample size of the original cohort (N = 884) was calculated on the basis of prevalence of secondary hyperparathyroidism, expected to occur in approximately 40% of patients with stage 4 CKD over a 3-year follow-up period; patients with stage 4 CKD account for approximately 17% of the sample population on the basis of an appropriate questionnaire previously completed by participating Nephrology units. We estimated that 1,000 subjects would have allowed for an estimate of a 95% confidence interval for the frequency of each outcome in the most restrictive condition (50%) with ±1.6% precision, and that this sample would have allowed to examine also the results from each CKD stage.

**Fig 1:**
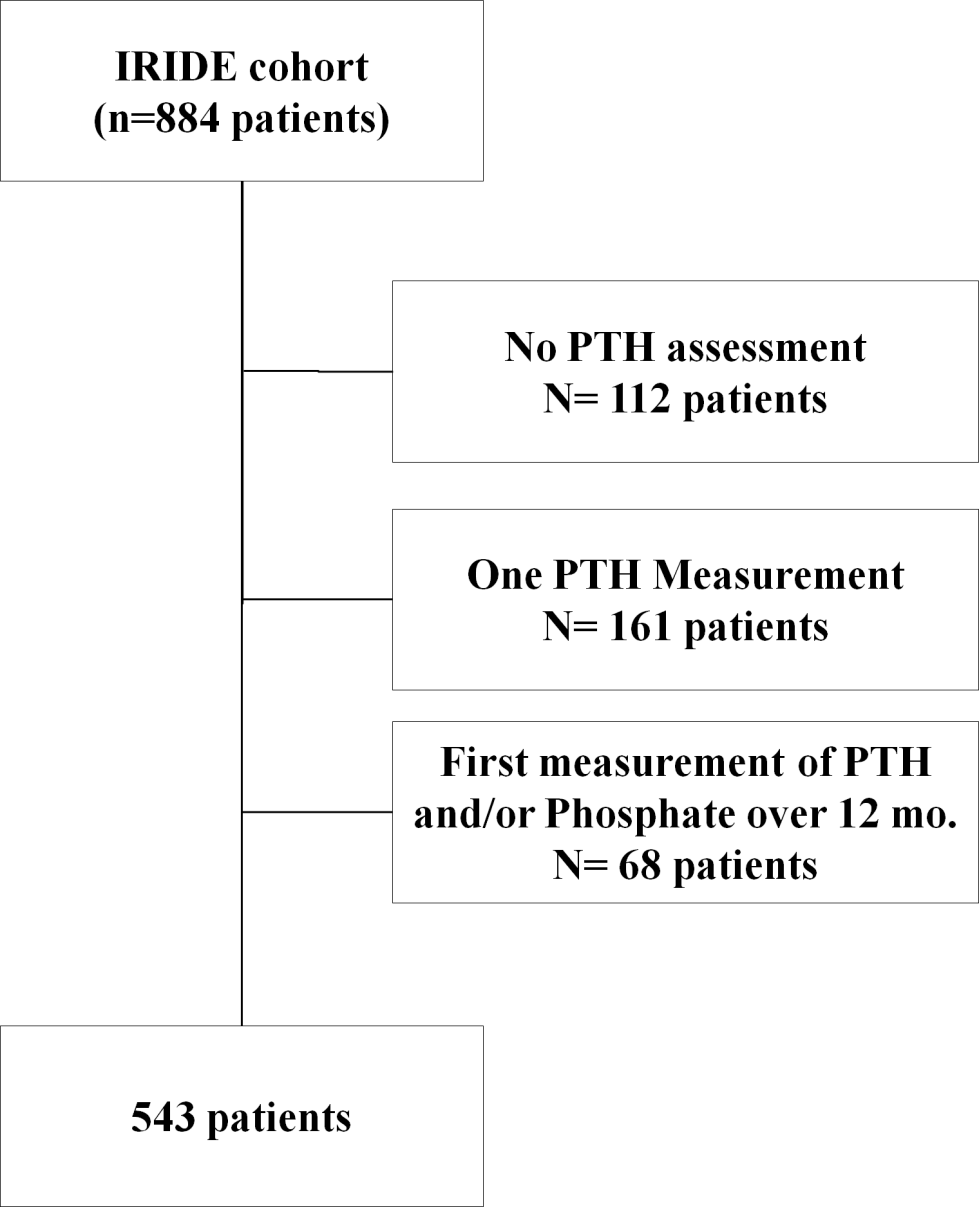
Selection algorithm of patients.

Out of the original IRIDE cohort (N = 884 patients), we selected 543 patients with at least two measurements of serum PTH within the first year (Fig 1).

### Data collection

According to IRIDE protocol, data were collected in each participating center, starting from baseline visit, every 6 months, for 36 months (for a maximum of 7 visits). At baseline visit, Nephrologists collected demographics data, medical history, laboratory test (including proteinuria) and therapy data. The medical history included diagnosis of hypertension according to K/DIGO guidelines (Systolic BP>130 and/or Diastolic BP>80 with proteinuria and/or diabetes) [13] or in antihypertensive treatment with at least one anti-hypertensive drug, diabetes (either self-reported diagnosis, use of hypoglycemic drugs, or a fasting glucose level>126 mg/dL), cardiovascular disease (CVD) (defined as electrocardiography-documented angina, history of myocardial infarction, stroke, transient ischemic attack, intermittent claudication, and prior revascularization procedures) and dyslipidemia (serum Cholesterol>200 mg/dL and/or use of statin). Laboratory parameters were measured by standard methods in the clinical laboratories of participating centers. Estimated GFR (eGFR) was calculated by the CKD-EPI (CKD Epidemiology Collaboration) creatinine equation; creatinine was not standardized to isotope-dilution mass spectrometry values and we therefore reduced creatinine levels by 5%, according to Skali et al [14].

Serum PTH was measured with the Allegro intact PTH assay from Nichols Institute Diagnostics Inc (72.4%). The values of PTH assessed with other method (PTH immunoradiometric assay by Scantibodies laboratories Inc.) were standardized by equations of the weighted Deming regression lines [15]. Proteinuria measured using the 24 hour collection (N = 481). Serum Ca was corrected for albumin levels.

### PTH change and outcome

Our aim was to assess the association between change of serum PTH values from baseline and risk of renal progression. Longitudinal changes of serum PTH were calculated as the absolute difference between all available PTH values after the first assessment and the basal values (∆PTH). In each single patient, the basal value of serum PTH was considered that measured at the first assessment within the first 12 months of FU. For each subject, ΔPTH was initially equal to 0 and was updated at each successive PTH assessment.

For example, if the patient had 3 evaluations at baseline and at 6 / 24 and 30 months, respectively equal to 100, 100, 120 and 70pg/mL, our variable of interest (∆PTH) was 0 for six months, +20 in the following 18 months and -30 until end of follow up. Therefore for this patient, three ∆PTH (0, +20 and -30) and corresponding intervals (6, 18 and 6 months) were taken into account in the models.

Primary outcome in this sub-analysis was renal death, defined as a composite endpoint including ESRD (chronic dialysis or pre-emptive kidney transplantation) or all-cause death before ESRD, whichever occurred first. Patients were followed up from the day of baseline visit until September 30, 2014 ESRD or death before ESRD or for lost to follow-up censored on the date they had the last clinic visit. Local investigators contacted by phone patients missing two or more planned visits to ascertain vital status and start of ESRD. This latter event was subsequently confirmed by Regional Dialysis Registries.

### Statistics

Continuous variables were reported as either mean and standard deviation (SD) or median and interquartile range (IQR) according to their distribution (assessed by using Shapiro-Wilk test). Intergroup comparisons were performed by means of one-way analysis-of-variance or Kruskal-Wallis test, as appropriate. Categorical variables were expressed as percentages and compared by Chi-square test. To examine changes over time of laboratory values, we used a within-subjects ANOVA test, whereas for non-parametric variables we assessed the changes using Skillings–Mack test, that is a general Friedman-type statistic that can be used in almost any block design with an arbitrary missing-data structure [16].

A Generalized Linear Mixed Model with both fixed and random effects was fitted in order to assess which baseline variables were associated with changes of serum PTH over time (ΔPTH), using the compound symmetric as covariance structure, i.e. assuming that there is a correlation among separate measurements and that the correlation is constant regardless of how far apart the measurements are [17].

To estimate the role of baseline PTH and its longitudinal changes over time on the renal death multivariable Cox proportional hazards model was used to estimate hazard ratio (HR) and 95% confidence interval (CI). Model was adjusted for baseline covariates (age, gender, diabetes, prior CV disease, hypertension, RAS inhibitors, serum total calcium and phosphate, PTH and proteinuria) identified *a priori* as possible confounders and stratified by CKD stages and cohort dimension. Then, we performed a model with the same baseline covariates, but adding ΔPTH, as both continuous and discrete (ΔPTH quartiles) time-varying dependent covariates. Indeed Cox model allows to analyze covariate information that change over time, with the hazard proportional to the instantaneous probability of an event at a particular time [18].

To analyze non-linear association between continuous ΔPTH and renal death restricted cubic spline according to Harrell method was used [19].

A two-tailed P value <0.05 was considered significant. Data were analyzed using STATA 11.2 (College Station, Texas USA).

## Results

As illustrated in Fig 1, 543 patients were included into the analysis and 341 patients were excluded. Of note, the incidence rate of renal death in those patients excluded from this analysis were not different than those included, respectively 6.4 (CI 95%: 4.9–8.2) events per 1000 person-year *vs* 6.3 (CI 95%: 5.2–7.6) events per 1000 person-year (P = 0.940).

Demographic and clinical characteristics stratified by CKD stage are illustrated in Table 1. Briefly, whole population (all white) was characterized by advanced age (46.8% of patients were aged 65–80 years and 15.5% over 80 years). Males were the majority of the population (58.4%). At baseline, eGFR was in median 36 (IQR: 22.4–56.8) mL/min/1.73 m^2^ and proteinuria was 0.4 (0.1–1.1) mg/24h. The distribution by CKD stages was 21% in stage 1–2, 17% in stage 3a, 22% in stage 3b, 28% in stage 4 and 12% in stage 5. As expected, from earlier to more advanced stages of CKD, prevalence of hypertension, anemia and CVD progressively increased as the serum levels of PTH and phosphate. Hypertensive patients were all treated with at least one anti-hypertensive drug.

**Table 1:** Demographics and basal clinic features of study cohort overall and by CKD stage.

	Overall	Stage 1–2	Stage 3a	Stage 3b	Stage 4	Stage 5	P
Number (%)	543 (100)	115 (21.2)	92 (16.9)	117 (21.6)	151 (27.8)	68 (12.5)	
GFR-EPI (mL/min/1.73 m^2^)	36(22–57)	75(67–93)	51(48–55)	36(32–41)	23(19–26)	12(10–15)	
Age (years)	66.3±15.4	56.9±17.8	69.7±9.5	69.7±11.7	69.6±13.3	64.5±15.4	<0.0001
Male gender (%)	58.4	59.1	59.8	61.5	58.9	48.5	0.506
Diabetes (%)	27.8	24.4	33.7	28.2	29.8	20.6	0.361
CVD (%)	23.8	9.6	28.3	20.5	31.8	29.4	<0.0001
Dyslipidemia (%)	41.6	34.8	42.4	41.0	45.7	44.1	0.322
Hypertension (%)	93.7	87.0	92.4	95.7	96.7	97.1	0.009
ACEi and/or ARBs (%)	54.0	63.5	55.5	56.4	56.3	26.5	<0.0001
Anemia (%)	26.9	3.5	10.9	17.1	40.4	75.0	<0.0001
Proteinuria (g/day)	0.4 (0.1–1.1)	0.2 (0.1–0.9)	0.2 (0.0–0.4)	0.3 (0.1–0.9)	0.5 (0.2–1.1)	0.7 (0.4–1.5)	<0.0001
Serum Total Calcium (mg/dL)	9.3±0.6	9.3±0.5	9.4±0.5	9.3±0.5	9.2±0.6	9.2±0.6	0.061
Serum Phosphate (mg/dL)	3.7±0.8	3.4±0.6	3.3±0.7	3.3±0.5	3.9±0.7	4.5±0.9	<0.0001
Serum PTH (pg/mL)	46 (28–81)	28 (18–39)	38 (26–55)	46 (35–67)	63 (43–102)	90 (54–151)	<0.0001

Pharmacologic treatment of CKD-MBD over CKD stages at baseline are reported in Table 2. Notably, prescription of nutritional Vitamin D did not change across CKD stages, whereas use of activated vitamin D progressively increased up to 4-fold from earlier to advanced CKD stages. Similarly, the use of calcium salt and non-calcium containing phosphate binders (lanthanum carbonate and sevelamer) increased with advancing of renal disease.

**Table 2:** Therapeutic features of CKD-MBD of study cohort, overall and by CKD stage, at baseline.

	Overall	Stage1-2	Stage 3a	Stage 3b	Stage 4	Stage 5	P
Cholecalciferol (%)	16.4	20.0	19.6	15.4	16.7	7.4	0.206
Vitamin D analogue (%)	46.6	20.0	32.6	43.4	62.9	79.4	<0.0001
Calcidiol(%)	1.6	0	0	3.9	1.1	1.9	
Calcifediol(%)	21.8	47.8	43.3	23.5	15.8	7.4	
Calcitriol (%)	57.3	39.1	36.7	62.7	60.0	66.7	
Paracalcitol(%)	19.4	13.0	20.0	9.8	23.2	24.1	
Calcium supplement (%)	17.7	10.4	4.4	13.7	25.2	38.3	<0.0001
Non-Calcium-Phosphate binders (%)	5.7	0	0	0.9	8.6	26.5	<0.0001
Aluminium based (%)	1.4	0	0	0.9	4.6	8.8	
Sevelamer (%)	2.6	0	0	0	2.0	11.8	
Lanthanium (%)	1.8	0	0	0	2.0	6.0	

### Survival analysis

During follow-up (median 36 months IQR: 33–37 months), no significant difference in serum levels of PTH was detected (p = 0.08). In these patients we registered a reduction of eGFR (p<0.0001) and a raise of serum phosphate (p<0.0001) in the absence of changes of serum calcium. ΔPTH over time was in median 0 (IQR: -18/18) pg/mL. The generalized linear mixed model showed that higher serum phosphate, CKD stages 3b-5 and lower serum PTH at baseline were associated with an increased risk to have a positive ∆PTH over time (Table 3).

**Table 3:** Basal predictors of change over time in serum PTH (ΔPTH) by general linear mixed model.

	Beta (C.I. 95%)	P
Time (months)	0.06 (-0.38; 0.24)	0.673
Age (years)	0.12 (-0.24; 0.48)	0.517
Male gender (yes vs no)	4.87 (-5.0; 14.75)	0.333
Diabetes (yes vs no)	7.40 (-3.19; 17.98)	0.171
CKD stage **1–2**	Ref	
Stage 3a	3.93 (-11.87;19.73)	0.626
**Stage 3b**	**15.35 (0.64; 30.06)**	**0.041**
**Stage 4**	**35.14 (19.78–50.49)**	**<0.0001**
**Stage 5**	**58.95 (38.12–79.79)**	**<0.0001**
Hypertension (yes vs no)	1.64 (-18.7; 22.0)	0.874
Prior CVD (yes vs no))	7.28 (-4.49; 19.05)	0.225
**Serum Phosphate (mg/dL)**	**10.09 (2.78;17.40)**	**0.007**
Serum total Calcium (mg/dL)	-6.35(-14.89; 2.19)	0.145
**Serum PTH (pg/mL)**	**-0.38 (-0.43;-0.33)**	**<0.0001**
Proteinuria (mg/day)	1.44 (-2.98; 5.85)	0.523
Constant	34.5 (-52.87; 121.91)	0.439

During follow-up we registered 114 renal deaths (82 ESRD events, all initiation of chronic dialysis, and 32 overall-cause deaths before ESRD). Incidence rates were 4.5 per 1000 patient year (95% CI, 3.6 to 5.6) for ESRD and 1.8 per 1000 patient-year (95% CI, 1.3 to 2.6) for death before ESRD.

Cox analysis showed that basal serum levels of PTH and phosphate and proteinuria were main modifiable predictors of renal death (Model 1 in the Table 4). When we added to the model containing baseline variables ΔPTH quartiles (Model 2 in Table 4), as discrete time-varying covariate, we found a graded association with the risk of renal death that became significant for the highest ΔPTH quartile (>18 pg/mL; P = 0.026).

**Table 4:** Cox Analysis of determinants of renal death adjusted for baseline covariates (model 1) and adding time varying covariate (model 2: Model 1 + delta PTH) stratified by CKD stage and cohort dimension.

	Model 1		Model 2	
	Hazard Ratio (95% C.I:)	P	Hazard Ratio (95% C.I.)	P
Age (years)	1.01 (0.99–1.03)	0.420	1.01 (0.99–1.03)	0.447
**Males vs Females**	**1.92 (1.17–3.13)**	**0.010**	**1.90 (1.16–3.12)**	**0.010**
Diabetes Yes vs No	1.21 (0.75–1.96)	0.445	1.25 (0.77–2.03)	0.373
**CVD Yes vs No**	**1.83 (1.15–2.92)**	**0.011**	**1.86(1.17–2.96)**	**0.009**
Hypertension Yes vs No	0.88 (0.20–3.87)	0.855	0.88 (0.20–3.88)	0.866
RAS* inhibitors Yes or No	0.81 (0.49–1.32)	0.409	0.72 (0.44–1.20)	0.219
**Proteinuria (+1 g/day)**	**1.35 (1.14–1.61)**	**0.001**	**1.31 (1.10–1.55)**	**0.002**
**Serum Total** Calcium (+1 mg/dL)	0.84 (0.57–1.25)	0.388	0.87 (0.59–1.28)	0.465
**Serum Phosphate** (+1 mg/dL)	**1.46 (1.07–1.98)**	**0.015**	**1.39 (1.03–1.89)**	**0.035**
**Serum PTH (+10 pg/ml)**	**1.03 (1.01–1.04)**	**<0.0001**	**1.04 (1.02–1.05)**	**<0.0001**
**ΔPTH quartiles** **< -18** **-18 to 0** **0 to 18** **>18 pg/mL**	**-** **-** **-** **-**		**Ref.** **1.09 (0.47–2.55)** **1.70 (0.74–3.89)** **1.91 (1.08–3.38)**	**-** **0.820** **0.201** **0.026**

*RAS, Renin Angiotensin System

**Fig 2:**
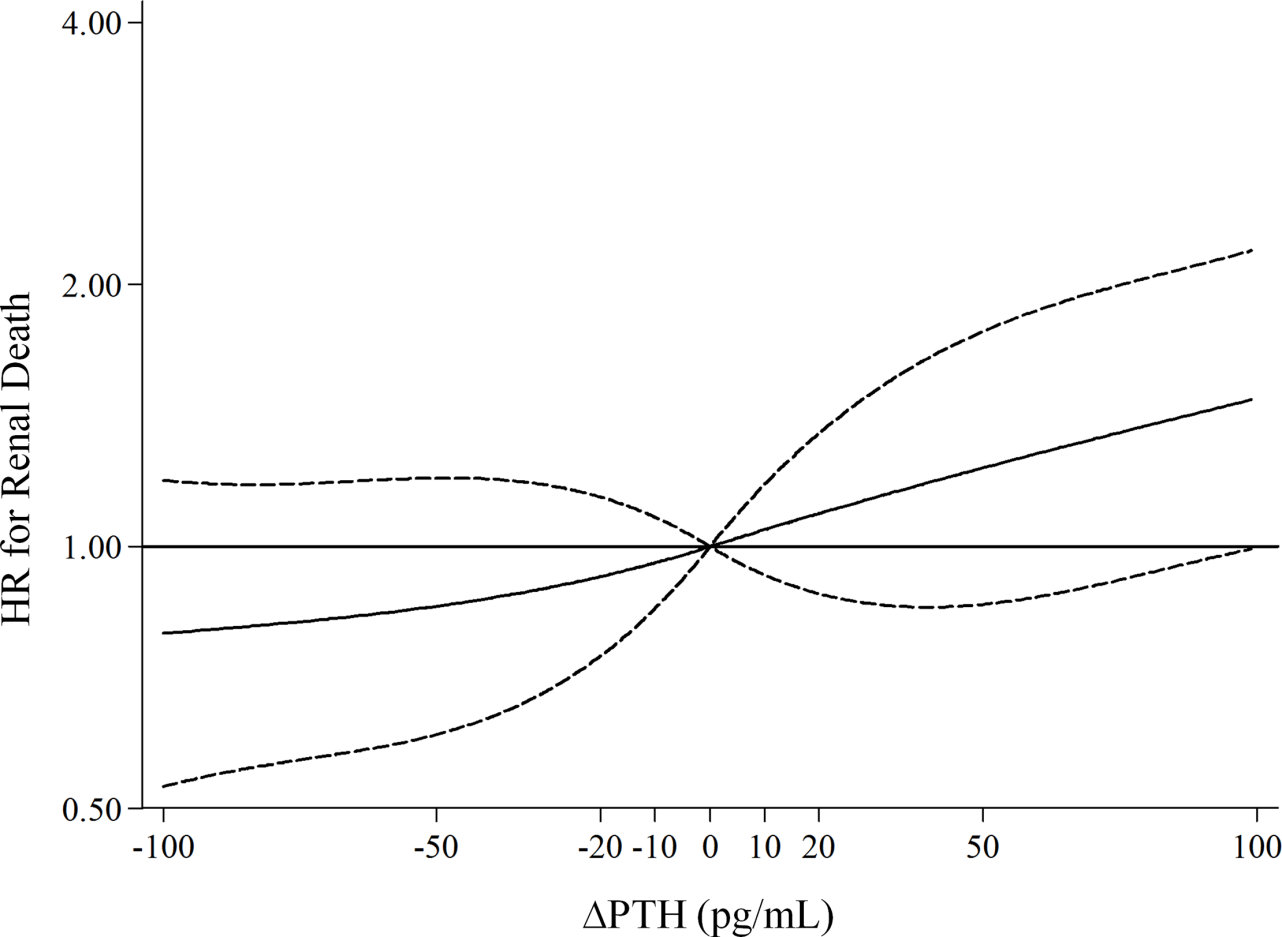
Plot of adjusted hazard ratio and 95% confidence intervals (as indicated by the curvilinear dash lines; the horizontal lines represent HR = 1) for renal death by level of ∆PTH as continuous variable (reference level, 0 pg/mL).

The importance of PTH changes over time was confirmed when using ΔPTH as continuous time dependent variable. Restricted cubic spline with four knots showed that HR for renal death increased in a linear fashion with the increment in ΔPTH (Fig 2; P value for non-linear association: 0.805) and linear ΔPTH was significantly associated with renal death (HR:1.02; C.I.:1.01–1.04 for every 10 pg/mL; p = 0.004).

We performed a sensitivity analysis by excluding CKD stage 1–2 from Cox analysis. In this sub cohort (N = 428 patients), the risk for renal death was not different from that observed in whole cohort (HR:1.04; C.I.:1.02–1.06 for every 10 pg/mL; p<0.001). Finally, we performed a further sensitivity analysis showing that ∆PTH was independently associated with ESRD alone (HR for ESRD: 1.02 95% C.I.: 1.00–1.04; P = 0.019).

## Discussion

In a cohort of 543 patients in stage 1–5 CKD regularly followed in 24 Italian Nephrology units we found that serum increment over time of PTH heralds worst renal outcome. More specifically, we examined the association between renal death and the longitudinal changes within the same subject of serum PTH with respect to baseline value (∆PTH). In particular, we observed that risk progressively rose, as ∆PTH increased, independently from the well-known cardio-renal risk factors related to CKD.

These findings are relevant for two main reasons. First, in ND-CKD patients serum PTH may change frequently due to the deterioration of kidney function over time and unmodified phosphate intake, as GFR deteriorates. In fact, to maintain homeostasis of phosphate, renal function of the diseased kidney must undergo adaptive changes, through the decrement in tubular reabsorption of phosphate, that prevent a rise in the serum phosphate until advanced deterioration of renal function occurs [1]. However, as Bricker proposed in his “trade-off hypothesis" [20], a biologic price is paid for these adaptive changes, with the development of secondary hyperparathyroidism that is mainly caused by phosphate retention. Furthermore, nephrologists' management of CKD-MBD includes the implementation of low protein and phosphate diet and the use of drugs, such as analogues of vitamin D, phosphate binders and calcium salt, that affects greatly serum PTH. Because our study was carried out in tertiary care, this aspect becomes essential, as evidenced by large use of CKD-MBD therapeutic agents at baseline (Table 2).

In a similar scenario, the use of statistical methods for time-to-event data that can incorporate time-dependent covariates is mandatory [21]. In particular, time-dependent Cox models represent a more appropriate tool to evaluate the longitudinal prognosis in chronic patients, since this model offers the opportunity to associate changes in exposure over time (time varying covariate) with outcome. Therefore this model can properly answer the critical question on how longitudinal changes in serum PTH versus baseline can affect the prognosis [22,23].

To our knowledge, this is the first time that this statistical method was used to assess the effect of longitudinal PTH changes in ND-CKD patients. A similar method was used by Kalanthar-Zadeh et al. in a cohort of about 58,000 patients on maintenance HD [23]. Those authors examined the associations between survival and changes in blood concentrations of quarterly mean PTH (as 8 categories: <100, >700 pg/mL and six 100 pg/mL groups in-between), using either time-dependent Cox models with repeated measures or fixed-covariate Cox models with only baseline values [23]. We have similarly examined the effect on renal death of fixed covariates measured at baseline and then adding ∆PTH, as both continuous and discrete (quartiles) time-dependent covariate.

When we considered fixed covariates measured at baseline, we found that higher serum PTH associated with an increased risk of renal death, as already reported in diverse ND-CKD cohorts, [5–8, 24–31]. The novel finding of our study is that when time-varying ∆PTH was added into the model, the longitudinal increment of serum PTH acted as a predictor of renal death independent of basal fixed covariate, i.e. older age, CKD stage, serum levels of PTH and phosphate, and, more important, independently from proteinuria, that is considered the main risk factor of CKD progression [32].

Noteworthy, we found that an increment of serum PTH over time was predicted by elevated levels of serum phosphate and more advanced CKD stages. These findings are consistent with pathogenic hypothesis that higher levels of serum phosphate stimulates directly the PTH secretion, as GFR decreases [33]. Furthermore, as previously reported, serum PTH levels rose from stage 1–2 to stage 5 [2–4]. Nevertheless early alterations of serum PTH are seldom detected at these stages of CKD and no recommendations are given for its management and prevention [9]. Indeed, we did not register any outcome in earlier stages suggesting that at these levels of CKD the effect on renal prognosis is probably of minor entity, as confirmed by sensitivity analysis in a sub-cohort of patients with GFR < 60 mL/min/1.73 m^2^. However, the relatively low number of renal outcomes in our study may be consequent to a limited follow up; indeed, previous work in a large community without renal insufficiency has reported that elevated plasma levels of PTH can account for 20% of the population-attributable risk proportion for CV mortality, indicating that elevated serum PTH may have a significant prognostic effect even in the absence of renal dysfunction [34].

Noteworthy, CKD patients with low and stable PTH levels may have Adynamic Bone Disease (ABD), that is associated with higher CV risk possibly dependent on increased vascular calcifications [35]. This is in apparent contrast with our findings. However, ABD is more common in dialysis patients, especially in those with advanced age, diabetes and undergoing high calcium load. On the other hand, low rate of death observed in the 3 years of FU did not allow to assess adequately this specific issue.

This study has several limitations. First, the observational design does not allow an interpretation of results in causal terms and we cannot draw any conclusion about the role of treatment on the PTH change. Second, we cannot generalize our findings to unreferred patients or to ethnic groups other than Caucasians. However the criteria to select CKD patients in stages 1–5 from Nephrology units distributed over almost the entire Italian territory, may be considered representative of Italian CKD population. Third, we cannot exclude survival bias that may be present in referred cohorts and selection bias (we excluded about 38% of original cohort); though incidence rates of renal death of patients excluded were not different than those included into the present analysis. Fourth, the cohort is made for 63% of elderly patients (>65 years) that may greatly affect the renal prognosis of these patients [30]; however, this cohort is a real picture of CKD population referred to nephrologists [29–31]. Finally, data on protein and phosphate intake were not collected [36].

In conclusion, the increment in serum PTH over 36 months represents an independent risk factor for renal death in ND-CKD patients. This finding provides a formal support to current guidelines that indicate the need of repeated measurements of serum PTH to better stratify the risk of CKD patients. Our data also highlight the importance of implementation in Nephrology research of statistical models with time dependent covariates in order to better define the prognosis of CKD patients [37]

## Supporting information

S1 dataset Complete dataset. (XLSX)
